# 4,5-Diamino-2-Thiouracil-Powered Dual-Mode Biosensor for Sensitive, Nonenzymatic Determination of Saliva Uric Acid Levels

**DOI:** 10.1155/2024/9944426

**Published:** 2024-09-25

**Authors:** Zipeng Wu, Lingyan Cheng, Shuhua Cai, Baochang Su, Yaowei Chen, Chunzong Cai, Weijin Guo, Dong Ma, Xin Cui

**Affiliations:** ^1^ Key Laboratory of Biomaterials of Guangdong Higher Education Institutes Department of Biomedical Engineering Jinan University, Guangzhou 510632, China; ^2^ Transfusion Department The First Affiliated Hospital of Jinan University, Guangzhou 510632, China; ^3^ Department of Biomedical Engineering Shantou University, Shantou 515063, China

**Keywords:** 4,5-diamino-2-thiouracil, colorimetric, nonenzymatic, photothermal, uric acid

## Abstract

Nonenzymatic and rapid monitoring of uric acid levels is of great value for early diagnosis, prevention, and management of oxidative stress–associated diseases. However, fast, convenient, and low-cost uric acid detection remains challenging, especially in resource-limited settings. In this study, a novel and rapid biosensing approach was developed for the simultaneous visualization and quantification of uric acid levels by using the unique surface plasmon resonance and photothermal property of 4,5-diamino-2-thiouracil (DT)-capped gold nanoparticles (AuNPs). With the presence of uric acid, DT-capped AuNPs rapidly aggregated, and a visible color/photothermal change was used for uric acid quantification within 15 min. The limit of detection was determined to be 11.3 and 6.6 *μ*M for the dual-mode biosensor, leveraging the unique structure of DT to optimize reaction kinetics. Moreover, the sensor exhibited excellent anti-interference capabilities and demonstrated potential for detecting a wide range of uric acid concentrations in complex samples, thereby reducing the need for extensive sample dilution and complex material synthesis procedures. Furthermore, validation against gold standard testing indicates that this biosensor could serve as a highly sensitive assay for quantifying uric acid levels in point-of-care applications, particularly in resource-limited settings.

## 1. Introduction

Uric acid is one of the endogenous antioxidants and the final product of purine nucleotide catabolism in biological fluids such as serum, plasma, urine, sweat, and saliva [1, 2]. The excessive (> 420 *μ*M, hyperuricemia) or decreased (< 140 *μ*M, hypouricemia) levels of uric acid in human serum are an indicator of many diseases including gout, cardiovascular risk, renal failure, multiple sclerosis, Parkinson's disease, and many other disorders associated with oxidative stress [3–5]. It is well established that there is a strong relationship between serum and saliva uric acid levels in most cases, suggesting the potential of saliva uric acid determination as a noninvasive alternative for blood tests because saliva testing has the advantages of being simple, safe, and noninvasive as compared to the risk of infection induced by invasive blood sampling [6–9]. The normal range of saliva uric acid level is between 150 and 350 *μ*M. Thus, noninvasive and cost-effective estimation of saliva uric acid levels is expected to be helpful for daily diagnosis, treatment, and follow-up of hyperuricemia and gout.

Both enzymatic and nonenzymatic methods have been developed to quantify the uric acid levels. For enzymatic sensing of uric acid, they have high accuracy and sensitivity. Among them, electrochemical sensors feature high sensitivity, rapid response times, and facilitated integration into flexible wearable devices. Moraes et al. developed an electrochemical sensor using *o*-phenylenediamine (*o*-PD) for antibiofouling and uricase for saliva uric acid sensing on a screen-printed carbon electrode and achieved a limit of detection (LOD) of 18.7 *μ*M [10]. Kim et al. reported a mouthguard biosensor using an enzyme (uricase)-modified screen-printed electrode system for real-time saliva uric acid detection in a linear range of 0–1000 *μ*M [11]. However, they need expensive microfabrication manufacturing processes and power supply, are prone to surface contamination or corrosion of the electrodes for electrode systems, and have a short shelf life. Currently, various optical sensors have been developed for quantitative uric acid analysis. The traditional and widely used method relies on a chromogenic system based on 3,3′,5,5′-tetramethylbenzidine (TMB), H_2_O_2_, and horseradish peroxidase (HRP). When uric acid is oxidized by urease to produce H_2_O_2_, TMB undergoes catalytic oxidation to form oxTMB, turning the solution from colorless to blue [12]. This process establishes a relationship between UA concentration and solution absorbance. For example, Chi et al. introduced a copper nanozyme composed of a copper phosphate framework and organic components with embedded uricase, combining the two oxidation steps of uric acid and TMB in a single reaction. The colorimetric sensor exhibits a linear response range within 1–50 *μ*M [13]. Inspired by the catalytic center of HRP, Ma et al. developed an efficient peptide-based HRP mimic using a covalent co-assembly strategy. This approach demonstrated a broad detection range from 5 to 800 *μ*M with a lower LOD. However, its inherent color interference limits its application in optical sensors [14]. Although these enzymatic methods showed high sensitivity for saliva uric acid detection, it is inconvenient for daily personal usage because the storage of the expensive enzyme requires harsh conditions such as low temperature and precise control of pH [15].

Nonenzymatic sensing of uric acid has been developed using the high-performance liquid chromatography [16, 17], capillary electrophoresis [18], electrochemical oxidation [19, 20], molecular imprinting technology [21, 22], lanthanide metal complexes [23], metal–organic framework [24], hydrogen-bonding interaction [15], and fluorescence probes [25, 26]. They need no enzyme and harsh storage conditions for saliva uric acid detection. However, they often require sophisticated equipment and skilled sample preparation (e.g., high-performance liquid chromatography and capillary electrophoresis) and time-consuming and complicated material synthesis process (e.g., electrochemical oxidation, molecular imprinting, and metal–organic framework). Fluorescence and colorimetric approaches have been extensively explored and utilized due to their inherent advantages and specific applications in nonenzymatic sensing of uric acid [15, 25, 26]. Fluorescence-based biosensors harness the emission of light from fluorophores upon excitation by a specific wavelength of light. This method offers high sensitivity and selectivity, allowing for real-time detection and quantification of analytes down to the nanomolar level [27]. However, fluorescence-based biosensors often face challenges such as photobleaching of fluorophores under prolonged exposure to light, which limits their long-term stability and utility in continuous monitoring applications. Additionally, background fluorescence from biological samples can interfere with signal detection, requiring sophisticated signal processing techniques to enhance specificity. In contrast, colorimetric biosensors rely on changes in color intensity or wavelength absorption upon interaction with a target analyte [28]. This approach offers simplicity in signal readout and does not require complex instrumentation, making it suitable for cost-effective, point-of-care diagnostics and applications without the need for specialized training. Despite their advantages, colorimetric biosensors may lack the sensitivity of fluorescence-based methods, limiting their applicability to detecting low concentrations of analytes. Additionally, the colorimetric signal can be affected by environmental conditions such as pH and temperature, necessitating careful calibration and control [29]. Moreover, the detection ranges in current nonenzymatic sensing of uric acid are mostly narrow and within several or tens of *μ*M (Table S1), which requires a large-volume sample dilution for clinical applications. Hence, a stable, nonenzymatic, and economic approach with high specificity and broad detection range for rapid assessment of uric acid levels is highly desirable.

**Scheme 1:**
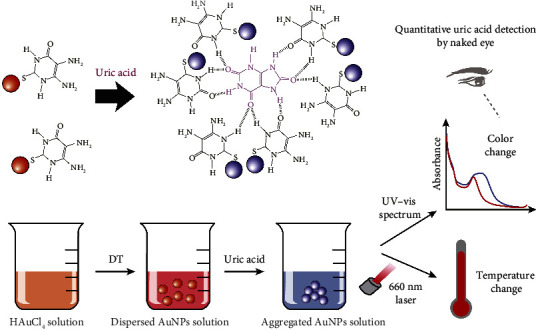
Schematic illustration of the nonenzymatic, dual-mode colorimetric/photothermal biosensing approach for rapid determination of uric acid levels. Gold nanoparticles (AuNPs) capped with 4,5-diamino-2-thiouracil (DT) were first synthesized and applied for quick uric acid recognition via possible hydrogen-bonding interaction, inducing a decreased distance among AuNPs which could be characterized by a visible color change using a UV–visible spectroscopy and a temperature change induced by the photothermal effects of AuNPs due to aggregation-induced plasmon resonance change.

In this study, we present a novel and dual-mode colorimetric/photothermal assay for the quantitative detection of uric acid levels within a range of several hundreds of micromoles by taking advantage of the novel 4,5-diamino-2-thiouracil (DT)-capped gold nanoparticles (AuNPs). When uric acid is present (Scheme 1), DT can quickly bind to uric acid via hydrogen-bonding interaction because of the unique structure of DT, thus considerably shortening the sample-to-result time to ∼15 min. Compared with the previous nonenzyme sensing strategy for uric acid quantification, the developed biosensor allows (1) economic but sensitive and specific uric acid detection, (2) dual-mode detection of uric acid in a wide working range of 6.6–250 *μ*M or 11.3–500 *μ*M, and (3) stable uric acid detection under various storage times (0–8 weeks) and pH values (5.5–9.5). Moreover, this assay does not require complicated material synthesis, large-volume sample dilution, lab environments, or technical training. To the best of our knowledge, this is the first report of DT-based nonenzymatic, dual-mode assay for the noninvasive and rapid detection of saliva uric acid levels without large volume of sample dilution.

## 2. Results and Discussion

### 2.1. Sensing Uric Acid Using DT-Capped AuNPs

Uric acid can form hydrogen bonds with an amide linkage via multiple hydrogen-bonding donor (–NH) and acceptor (–C=O) sites in uric acid [15, 30]. Inspired by these, we selected DT with the presence of two amine groups for hydrogen-bonding donor sites and investigated its performance for uric acid detection. When uric acid is present (Scheme 1), DT can quickly bind to uric acid via hydrogen-bonding interaction because of the unique structure of DT, inducing a decreased interparticle distance among AuNPs and a visible color change due to AuNPs aggregation–induced change in plasmon resonance wavelength. The presence of two amino groups in DT can potentially provide extra stabilization to the AuNPs and allow for increased opportunities for functionalization. This can lead to improved colloidal stability, thereby contributing to reliable and consistent biosensing performance, extending the shelf life, and enhancing the overall stability of the nanoparticles. To examine the possibility of hydrogen-bonding interactions between uric acid and DT, the molecular electrostatic potential (MEP) maps were created by using GaussView 6.0.16 in order to identify the electrophilic and nucleophilic sites of free uric acid and DT. Our results (Figure S1) showed that the uric acid possesses positive potential regions (*blue*) and a negative potential region (*red*) around the oxygen atoms can be observed in the DT, indicating the possible region from where electrophilic attack sites (electron acceptor) may interact with the nucleophilic sites (electron donor) [24].

**Figure 1:**
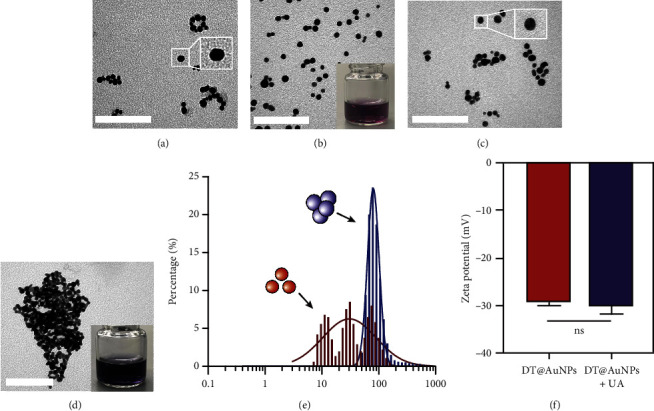
Characterization of the DT-functionalized AuNPs with the presence of uric acid. The transmission electron micrograph of (a) pure AuNPs, (b) pure AuNPs + PBS, (c) DT@AuNPs + PBS, and (d) DT@AuNPs + uric acid. (e) The dynamic light scattering (DLS) analysis for the AuNPs with the absence (red) and presence of 500 *μ*M uric acid (purple) after incubation for 10 min. (f) The zeta potential of nanoparticles showed the negative value with the presence of uric acid (500 *μ*M). The scale bar is 100 nm.

DT-capped AuNPs were synthesized by continuously adding NaBH_4_ (0.125%, w/v) solution into HAuCl_4_ (31.7 mM) and DT (1 mM) within 20 min under stirring. The successful conjugation of DT on AuNPs was confirmed by Fourier transform infrared spectroscopy (FTIR) spectral measurements (Figure S2). The absorption bands at 2550 cm^−1^ corresponding to S-H and the peaks around 1580 and 1054 cm^−1^ for the C=N and C-NH_2_ stretching vibrations confirmed the DT conjugation on the surface of the AuNPs via S–Au interaction [31]. To further confirm the successful conjugation of DT on AuNPs, transmission electron microscopy (TEM) was used, and the results in Figures 1(a) and 1(c) show that the AuNPs exhibit regular spherical shapes. Compared to the average particle size of 23 nm for bare AuNPs, the size of DT-capped AuNPs increased to 37 nm, and its surface morphology also changed, resulting in a much deeper color than bare AuNPs. This indicates that DT has been successfully modified on the surface of AuNPs.

Incubating uric acid (500 *μ*M) with DT-capped AuNPs caused obvious size increasing to 99.55 (±16.26) nm and a rapid red-to-blue color change within 10 min as shown in Figures 1(d) and 1(e), which is possibly induced by the hydrogen-bonding interaction between uric acid and the DT-functionalized nanoparticles [15, 30]. UV–visible spectrum demonstrated that a maximum surface plasmon band at 520 nm was observed in pure AuNPs, and a new intense surface plasmon band emerged from 520 to 620 nm due to the uric acid–induced aggregation of DT functionalized AuNPs (Figure S3), which were consistent with the results of TEM. Furthermore, the zeta potential study showed that DT-capped AuNPs were negatively charged (−29.23 ± 0.45 mV). After incubating with uric acid, the zeta potential value of AuNPs was slightly changed to −29.90 ± 1.69 mV (Figure 1(f)). All the experiments were performed in liquid environment with buffers at pH of 7.5 at room temperature.

### 2.2. Sensitivity of the DT-Capped AuNPs

**Figure 2:**
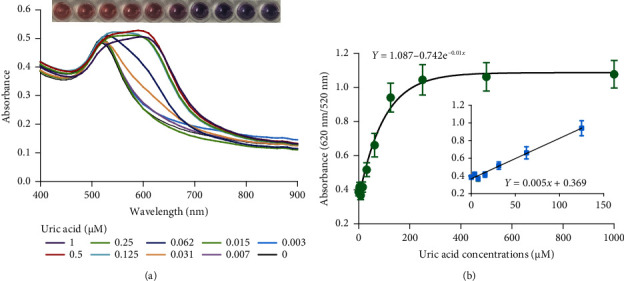
Performance of the DT-functionalized AuNPs for detecting uric acid. (a) UV–vis spectrums of DT-functionalized gold nanoparticles after incubation with different concentrations of uric acid in 1 × PBS buffer for 10 min, illustrating the concentration-dependent color change in AuNPs solution. (b) Relationship of uric acid concentrations with absorbance ratios (620 nm/520 nm) of AuNPs in 1 × PBS buffer. Each data point is calculated from at least three replicate measurements, and the error bars indicate the standard deviations.

The performance of the DT-capped AuNPs for quantitative uric acid quantification was investigated by detecting different concentrations of uric acid in 1 × phosphate-buffered saline (PBS) buffer at a pH of 7.5 at room temperature. As shown in Figure 2, a good exponential relationship (*R*^2^ = 0.96, *y* = 1.087 − 0.742e^−0.01*x*^) was observed between the ratios of absorption intensity (620 nm/520 nm) and the concentrations of uric acid in a range of 0–1000 *μ*M, which was much broader than the previously reported methods as shown in Table S1. The LOD was calculated as 11.3 *μ*M based on 3*σ*/*S*, where *S* is the slope of the linear calibration curve and *σ* is the standard deviations of the blank. Compared to previous nonenzyme sensing strategies (Table S1), our biosensor demonstrates a wide dynamic range from 0 to 250 *μ*M with a detection limit of 6.6 *μ*M to eliminate the large-volume sample dilution-induced detection errors. More importantly, this assay requires noncomplex procedures of material synthesis and can achieve naked eye–based uric acid detection.

### 2.3. Selectivity of the DT-Capped AuNPs for Uric Acid Quantification

**Figure 3:**
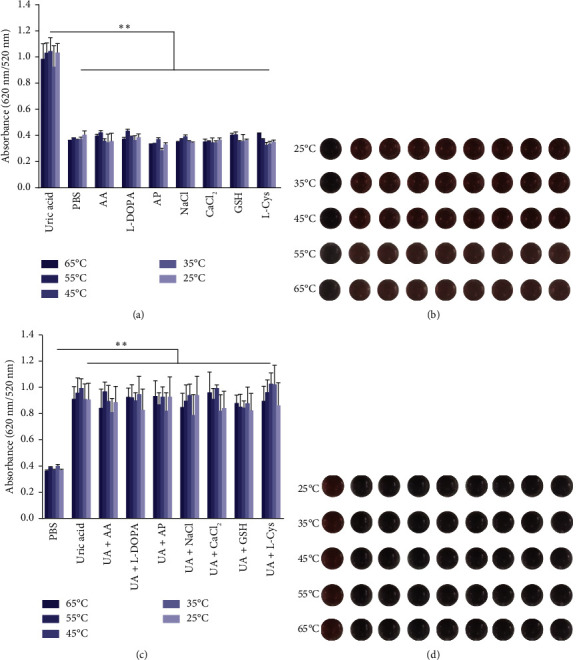
Colorimetric method based on DT-functionalized AuNPs for detection of uric acid. (a) Absorbance ratios (620 nm/520 nm) of DT-functionalized AuNPs with the addition of various interferents (250 *μ*M) including 4-acetamidophenol (AP), L-DOPA, ascorbic acid (AA), glutathione (GSH), L-cysteine (L-Cys), and metal ions Na^+^ and Ca^2+^ at different temperatures. Noted that no visual color changes were observed with or without the presence of various interferents. (b) Photos of DT-functionalized AuNPs with the addition of various interferents (250 *μ*M) at different temperatures. (c) Absorbance ratios (620 nm/520 nm) of DT-functionalized AuNPs after reaction with different mixtures of interfering substances (250 *μ*M) with uric acid (0.25 mM). Noted the distinct visible color (red) of AuNPs without the presence of uric acid. (d) Photos of DT-functionalized AuNPs after reaction with different mixtures of interfering substances (250 *μ*M) with uric acid (250 *μ*M) at different temperatures. ^∗∗^*p* < 0.01. Each data point is calculated from at least three replicate measurements, and the error bars indicate the standard deviations.

The selectivity of the DT-capped AuNPs for uric acid detection was evaluated in the presence of various interferents with similar structures including 4-acetamidophenol (AP), L-DOPA, ascorbic acid (AA), glutathione (GSH), L-cysteine (L-Cys), and environmentally relevant ions such as Na^+^ and Ca^2+^ at different temperatures (25°C, 35°C, 45°C, 55°C, and 65°C) but a consistent pH value of 7.5. As shown in Figures 3(a) and 3(b), no significant difference was observed within the ratios of absorption intensity (620 nm/520 nm) and the color of AuNPs among the various interferents (250 *μ*M) and that of the PBS group. Interestingly, the ratios of absorption intensity (620 nm/520 nm) with the presence of uric acid (250 *μ*M) likely decreased with the increase of temperature from 45°C to 65°C, which may be explained by the reduction of hydrogen-bonding strength induced by heating [32]. Moreover, we investigated the selectivity of the DT-capped nanoparticles for uric acid detection (250 *μ*M) in a mixture of different interfering substances (250 *μ*M), as shown in Figures 3(c) and 3(d). These results suggested that the DT-capped AuNPs were not interfered by these interfering substances, suggesting that the method was selective for rapid uric acid quantification.

### 2.4. Stability of the DT-Capped AuNPs for Uric Acid Quantification

**Figure 4:**
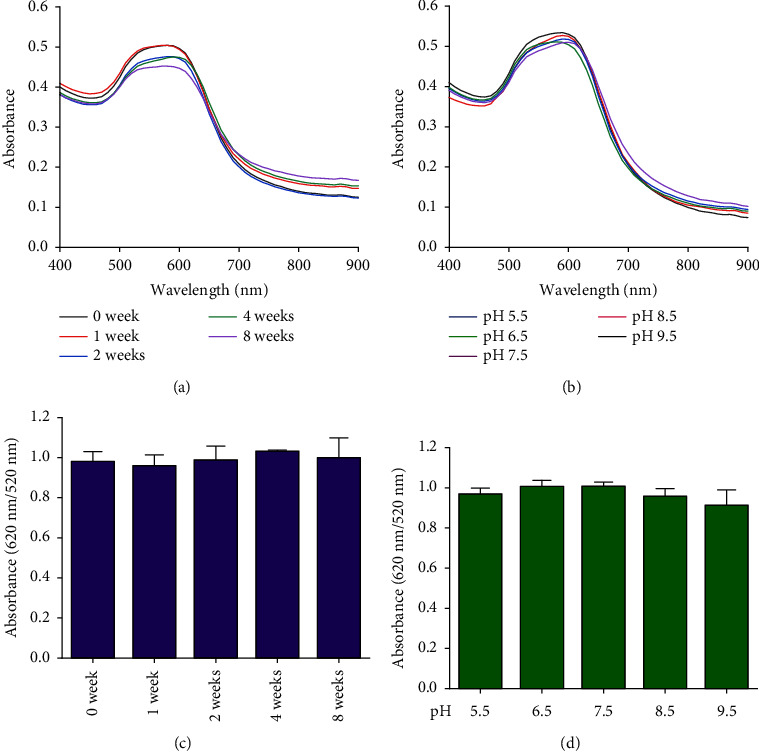
Stability of the DT-functionalized AuNPs for uric acid detection. (a) UV–vis spectral profiles of DT-functionalized AuNPs in the presence of uric acid (250 *μ*M) under various storage times (0, 1, 2, 4, and 8 weeks). (b) UV–vis spectral profile of DT-functionalized AuNPs in the presence of uric acid (250 *μ*M) at different pH values (5.5, 6.5, 7.5, 8.5, and 9.5). (c) Absorbance ratios (620 nm/520 nm) of DT-functionalized AuNPs under various storage times (0, 1, 2, 4, and 8 weeks). No significant difference in absorption intensity was observed with different storage times. (d) Absorbance ratios (620 nm/520 nm) of DT-functionalized AuNPs at different pH values. No significant difference in absorption intensity at 620 nm was observed among different pH values. Each data point is calculated from at least three replicate measurements, and the error bars indicate the standard deviations.

The stability of the DT-capped AuNPs at 4°C under various storage times (0, 1, 2, 4, and 8 weeks) and pH values (5.5, 6.5, 7.5, 8.5, and 9.5) was investigated (Figure 4). It was found that the nanoparticles showed little change in the absorption intensity at 520 nm/620 nm under various storage times from 4 to 8 weeks, as shown in Figures 4(a) and 4(b). Moreover, the nanoparticles at different pH values (5.5, 6.5, 7.5, 8.5, and 9.5) exhibited no significantly different responses to uric acid (250 *μ*M) (Figures 4(c) and 4(d)), which was possibly contributed by the two amine groups of DT inducing the improved stability of nanoparticles. Hence, the DT-capped AuNPs were stable at room temperatures for long-term storage and at different pH conditions for rapid uric acid analysis.

### 2.5. Photothermal Detection of Uric Acid Levels Using DT-Capped AuNPs

To further eliminate the requirement of spectrophotometers for rapid uric acid determination, we developed a portable and quantitative assay for uric acid detection by using the different photothermal effects of aggregated and nonaggregated AuNPs [33, 34]. AuNPs were irradiated with a laser (660 nm) after incubating with different concentrations of uric acid, and the temperature change during irradiation was monitored using a thermometer as the readout. Hence, the surface plasmon band changes of AuNPs with different levels of uric acid were converted into the photothermal-induced temperature change.

**Figure 5:**
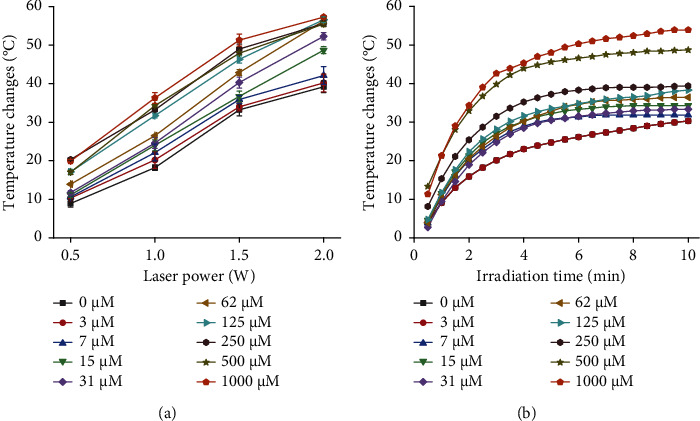
The impact of different laser (660 nm) irradiation power and duration on uric acid detection using DT-functionalized AuNPs. (a) Photothermal detection of uric acid levels under different irradiation laser (660 nm) power for 10 min. (b) Photothermal detection of uric acid levels under different irradiation times with a laser (660 nm) power of 1.5 W.

To investigate the effect of 660-nm laser on the photothermal detection of DT-capped AuNPs (Figure 5), we firstly examined the temperature changes of DT-capped AuNPs and uric acid sample mixtures under the irradiation of 660-nm laser for 10 min with different powers. The temperature changes for different levels of uric acid samples were similar for those lower than 31 *μ*M at the power condition of 0.5 W (Figure 5(a)). However, the temperature change at the power condition of 2.0 W was different, which was comparable for those uric acid samples with a level higher than 31 *μ*M. This suggests that too low or too high powers are unfavorable for triggering the photothermal effect of DT-capped AuNPs, which stems from the fact that DT-capped AuNPs recognize the low-concentration uric acid with less agglomeration. Similarly, when DT-capped AuNPs recognize a high concentration of uric acid with a high degree of agglomeration, the irradiation with a high-power laser will cause a saturated photothermal effect. A comparison of the temperature change among different concentrations of uric acid with a laser power of 1.0 and 1.5 W showed an obvious change at 1.5 W. Therefore, the photothermal detection of uric acid levels using DT-capped AuNPs was conducted at 660 nm with a power of 1.5 W.

Subsequently, the effects of different irradiation times on the temperature change in different concentrations of uric acid samples were evaluated using a 660-nm laser irradiation with a power of 1.5 W (Figure 5(b)). It was found that the temperature change in each sample reached the maximum value at 6 min, so the irradiation time of 6 min was selected for the following photothermal detection.

### 2.6. Saliva Uric Acid Quantification Using DT-Capped AuNPs

**Figure 6:**
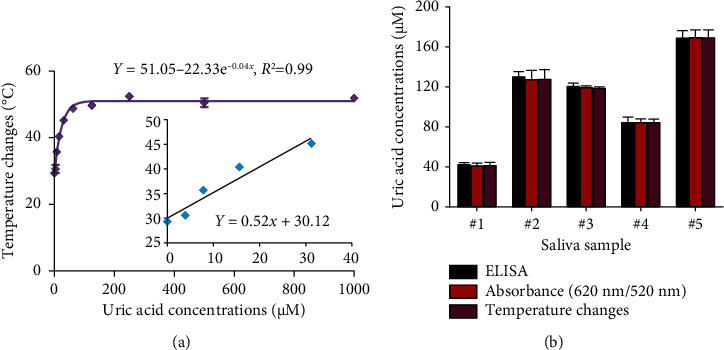
Photothermal detection of uric acid levels using DT-capped AuNPs in saliva solutions. (a) Calibration curves of temperature change and uric acid levels with a dilution factor of 7. (b) Photothermal and colorimetric detection of uric acid levels for real saliva samples. Note that a commercially available uricase-based enzymatic ELISA was used to quantify the uric acid levels in saliva samples for comparison. Each data point is calculated from at least three replicate measurements, and the error bars indicate the standard deviations.

The feasibility of the DT-capped AuNPs for clinical use was further evaluated by monitoring uric acid levels in saliva samples. A common approach to mitigate the matrix effects of numerous components of saliva is to dilute the sample [35]. Therefore, the saliva samples were filtered using a 3-kDa molecular weight cutoff (MWCO) microcentrifuge filter and further diluted using 1 × PBS buffer. We then investigated the effect of dilution factors of saliva samples on the performance of DT-capped AuNPs to detect different levels of uric acid using photothermal assays. As shown in Figure S4, with the increase of dilution factors, the matrix effect was weakened and reached the optimal state when the dilution factor is 7, whereas a good exponential (*R*^2^ = 0.99, *y* = 51.05 − 22.33e^−0.04*x*^) relationship exists between uric acid concentrations and the temperature change. The LOD was calculated as 6.6 *μ*M based on 3*σ*/*S* (Figure 6(a)). We further used the DT-capped AuNPs to quantify uric acid levels in five saliva samples with unknown uric acid levels. To compare the performance of the developed assay with the standard uric acid detection method, a commercially available uricase-based enzymatic ELISA was selected to quantify the uric acid levels in saliva samples by measuring the absorbance value at a characteristic absorption peak of 505 nm for H_2_O_2_-induced red quinones. The measured amounts using both colorimetric and photothermal approaches were in accordance with those using commercial enzyme-based ELISA detection (Figure 6(b)). Hence, these results demonstrated the potential clinical application for noninvasive and portable uric acid quantification in resource-limited areas without the requirement of bulky and expensive diagnostic equipment.

## 3. Conclusions

This work described a novel, nonenzymatic, cost-effective, and quantitative assay for noninvasive saliva uric acid monitoring using DT-capped AuNPs. To the best of our knowledge, this is the first study using DT-capped AuNP for effective uric acid quantification with high practicability because of the simple material synthesis, broad detection range, low sample dilution, and naked eye detection. Compared to previous nonenzyme sensing strategies, our biosensor offers several advancements. It combines economic feasibility with high sensitivity and specificity, achieving a detection limit of 11.3 and 6.6 *μ*M for colorimetric and photothermal modes, respectively. The biosensor also demonstrates a wide dynamic range within 500 *μ*M, making it suitable for diverse clinical applications. Moreover, it exhibits robust stability over storage times ranging from 0 to 8 weeks and under pH conditions spanning 5.5–9.5. Specially, this assay simplifies detection by eliminating the need for complex material synthesis, large-volume sample dilution, specialized laboratory environments, or extensive technical training. With further optimization of the detection sensitivity using novel nanomaterials with enhanced optical and chemical properties and integrating this assay with a portable smartphone spectrometer or miniaturized photothermal equipment, it enables rapid and accurate remote monitoring of uric acid levels without enzyme and large volume of sample dilutions, making it easy to conduct the test in resource-limited settings such as developing countries, rural hospitals, or community health service centers.

## 4. Materials and Methods

### 4.1. Materials and Reagents

All chemical compounds were used as received without further purification. Hydrogen tetrachloroaurate trihydrate (HAuCl_4_·3H_2_O) was purchased from GBCBIO Technologies Inc. (Guangzhou, China). DT, sodium borohydride (NaBH_4_), uric acid, AP, L-DOPA, ascorbate (AA), and 1 × PBS buffer were purchased from Macklin Co., Ltd. (Shanghai, China). Micro Uric Acid (UA) Content Assay Kit (BC1365) was purchased from Solarbio Science and Technology Co., Ltd. (Beijing, China).

### 4.2. Synthesis of AuNPs

AuNPs were synthesized according to a previously reported literature [15]. Briefly, HAuCl_4_ solution (0.5 mL, 31.7 mM) was mixed with DT (0.25 mL, 1 mM) and deionized (DI) water (23.5 mL), followed by adding the ice-cold reducing agent NaBH_4_ (2 mL, 0.125%) to the mixture under vigorous stirring for 20 min. The resulting wine-red-colored AuNP solution was stored in a refrigerator (4°C) and remained stable for at least 2 months.

### 4.3. Characterization of Nanoparticles

UV–vis spectra were examined with a modular multimode microplate reader (BioTek Synergy H1). Nanoparticle morphology and aggregation were characterized using a TEM (JEOL JEM-2010). The size and zeta potential of different samples were recorded with a Zetasizer Nano ZS Nanoparticle Size Analyzer (Malvern Instruments, Worcestershire, UK). FTIR was used to examine the DT-capped AuNPs by using a Nexus 670 FTIR spectrophotometer (Nicolet) in transmittance mode with KBr plates. DFT calculations were carried out with the Gaussian 09 D.01 package using the M06-2X exchange–correlation functional together with the def2-SVP25. The MEP maps were created by using GaussView 6.0.16 software.

### 4.4. Colorimetric and Photothermal Detection of Uric Acid

In a typical colorimetric assay, uric acid (20 *μ*L) in 1 × PBS buffer was gradually added to the DT-capped AuNPs (100 *μ*L) with various storage times (0, 1, 2, 4, and 8 weeks), followed by incubation at room temperature or different temperatures (25°C, 35°C, 45°C, 55°C, and 65°C) and pH values (5.5, 6.5, 7.5, 8.5, and 9.5) for 10 min and recording the UV–vis spectrum.

For photothermal detection, a 660-nm diode laser (LL664-2000 mw, Liangli Laser Corp., China) was used to irradiate the solution for another 6 min, and a digital thermometer probe TES-1310 (TES Electrical Electronic Corp., China) was used for monitoring the temperature change.

In a typical assay, 1 × PBS solution of uric acid (20 *μ*L) was gradually added to the AHM-capped AuNPs (100 *μ*L), followed by incubation at room temperature for 10 min. After recording the UV–vis spectrum, a 660-nm diode laser LL664-2000 mw (Liangli Laser Corp., China) was used to irradiate the solution for another 10 min, and a digital thermometer probe TES-1310 (TES Electrical Electronic Corp., China) was used for monitoring the temperature change. Note that all the experiments were conducted at room temperature with a pH value of 7.5, unless otherwise specified.

### 4.5. Pretreatment and Testing of Real Salivary Samples

All study participants signed consent forms according to JNU-approved institutional review board regulations. The saliva samples were filtered using a 3-kDa MWCO microcentrifuge filter to remove the impurities and diluted using 1 × PBS buffer. A similar measurement procedure was used for saliva uric acid sensing as that in 1 × PBS buffer. The samples were also tested using a commercially available uricase-based Micro Uric Acid Content Assay for comparison.

### 4.6. Statistics

Three independent experiments were repeated for each study. Statistical significance (^∗^, *p* < 0.05; ^∗∗^, *p* < 0.01) for multiple comparisons was examined by one-way ANOVA, followed by a Tukey analysis using Prism 7.0 (GraphPad Software, Inc.). Data were presented as mean ± SD.

## Data Availability

The data that support the findings of this study are available from the corresponding authors upon reasonable request.

## Supporting Information

Supporting Information Additional supporting information can be found online in the Supporting Information section. Figure S1: Electrostatic surface potential (ESP) maps from Gaussian-optimized structures of free uric acid, free DT, and their complex. Noted the favorable hydrogen-bonding interactions (arrow) in the complex of uric acid and DT. Figure S2: FTIR spectrum of AuNPs showed that the absorption bands disappeared at 2550 cm^−1^ corresponding to S-H after conjugating DT on AuNPs, which was possibly induced by the S–Au interaction. Figure S3: UV–vis spectrums of DT-functionalized AuNPs (red) after incubation with 500 *μ*M uric acid (purple). Figure S4: Photothermal detection of different levels of uric acid with different dilution ratios of saliva solutions (dilution ratios are × 1, × 3, × 5, × 7, and × 10). Table S1: Comparison of the nonenzymatic uric acid detection methods in recent years.
