# Supplementary material for: 4,5-Diamino-2-Thiouracil-Powered Dual-Mode Biosensor for Sensitive, Nonenzymatic Determination of Saliva Uric Acid Levels
Source: Int J Anal Chem. 2024 Sep 25;2024:9944426. doi: 10.1155/2024/9944426 (PMC11446619; doi:10.1155/2024/9944426)
Supplement: Supporting Information — Additional supporting information can be found online in the Supporting Information section. Figure S1: Electrostatic surface potential (ESP) maps from Gaussian-optimized structures of free uric acid, free DT, and their complex. Noted the favorable hydrogen-bonding interactions (arrow) in the complex of uric acid and DT. Figure S2: FTIR spectrum of AuNPs showed that the absorption bands disappeared at 2550 cm−1 corresponding to S-H after conjugating DT on AuNPs, which was possibly induced by the S–Au interaction. Figure S3: UV–vis spectrums of DT-functionalized AuNPs (red) after incubation with 500 μM uric acid (purple). Figure S4: Photothermal detection of different levels of uric acid with different dilution ratios of saliva solutions (dilution ratios are × 1, × 3, × 5, × 7, and × 10). Table S1: Comparison of the nonenzymatic uric acid detection methods in recent years. [file 9944426.f1.docx]

Supporting Information

**4,5-Diamino-2-thiouracil Powered Dual-Mode Biosensor for Sensitive, Nonenzymatic Determination of Saliva Uric Acid Levels**

Zipeng Wu^1,#^, Lingyan Cheng^1,#^, Shuhua Cai^1,#^, Baochang Su^2^, Yaowei Chen^1^, Chunzong Cai^1^, Weijin Guo^3,^*,Dong Ma^1,^*, Xin Cui^1,^*

1 Key Laboratory of Biomaterials of Guangdong Higher Education Institutes, Department of Biomedical Engineering, Jinan University, Guangzhou 510632, China

2 Department of blood transfusion, The First Affiliated Hospital of Jinan University, Guangzhou 510632, China

3 Department of Biomedical Engineering, Shantou University, Shantou 515063, China.

# These authors contributed equally to this work.

*Correspondence should be addressed to Xin Cui (cx2019@jnu.edu.cn), Weijin Guo (guoweijin@stu.edu.cn) and Dong Ma (tmadong@jnu.edu.cn)

# 1. Electrostatic surface potential (ESP) maps of pure DT and uric acid.


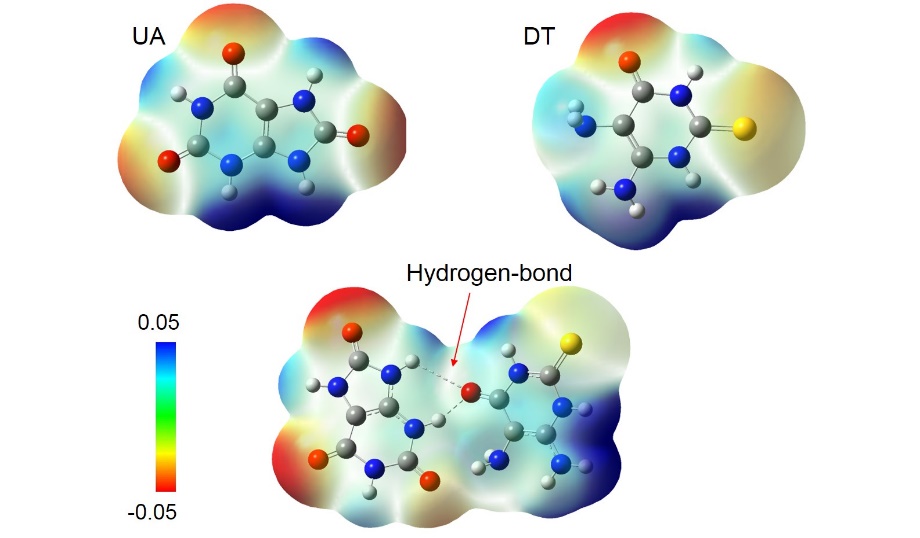


**Figure S1.** Electrostatic surface potential (ESP) maps from Gaussian optimized structures of free uric acid (UA), free DT and their complex. Noted the favorable hydrogen-bond interactions (*arrow*) in the complex of uric acid and DT.

# 2. FTIR spectrum of pure DT and DT functionalized AuNPs.


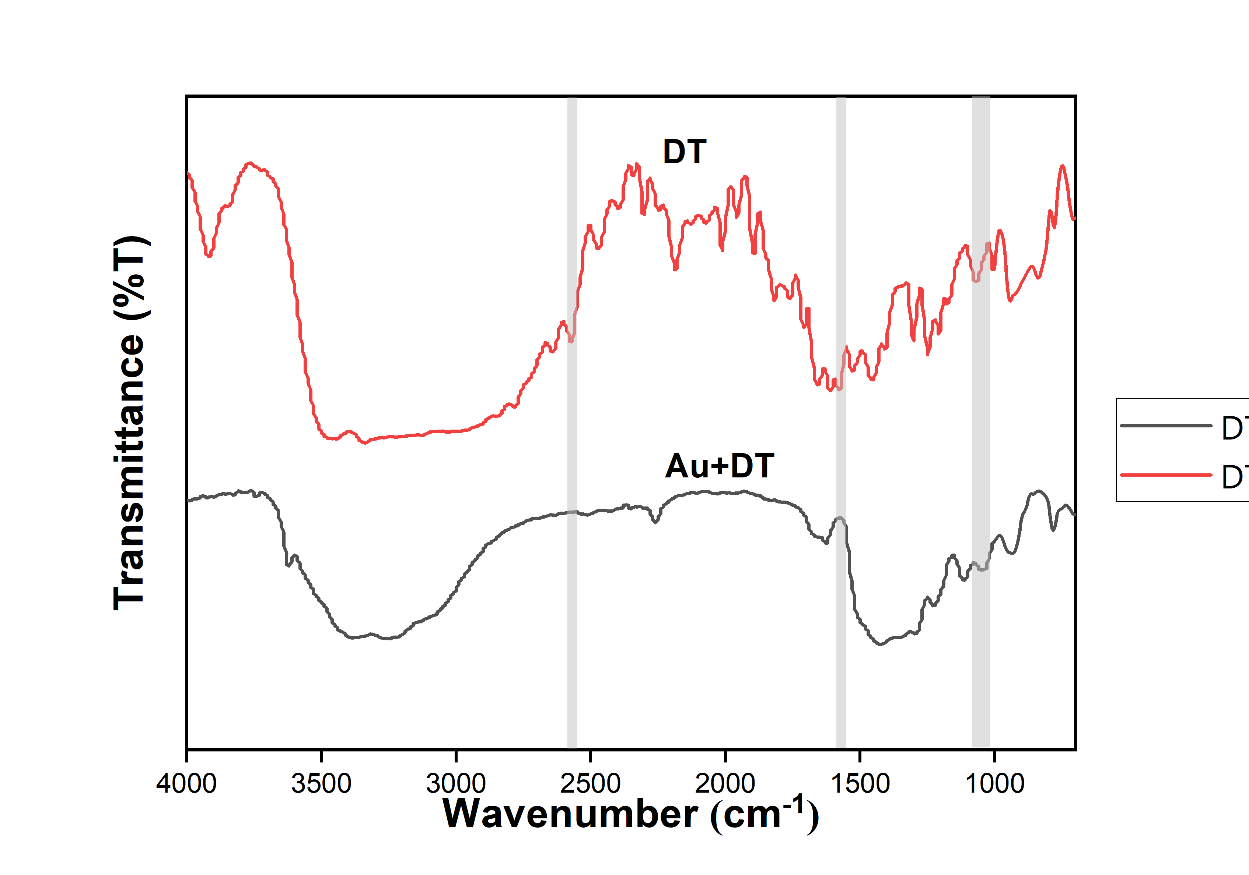


**Figure S2.** FTIR spectrum of gold nanoparticles shown that the absorption bands disappeared at 2550 cm^-1^ corresponding to S-H after conjugating DT on AuNPs, which was possibly induced by the S-Au interaction.

# 3. UV-vis spectrums of DT functionalized gold nanoparticles.


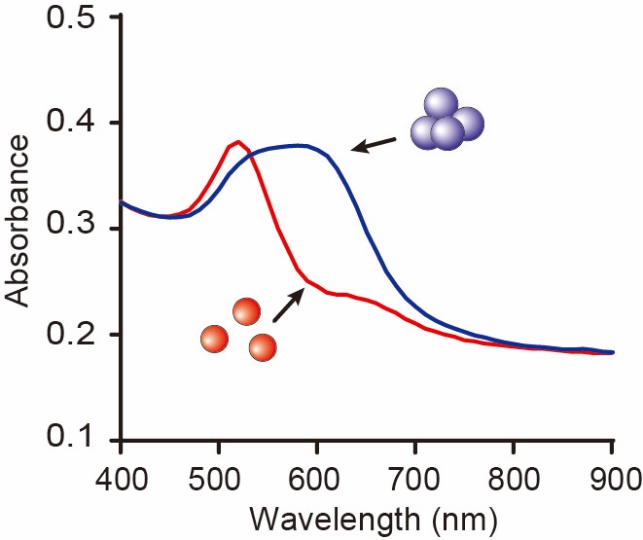


**Figure S3.** UV-vis spectrums of DT functionalized gold nanoparticles (*red*) after incubation with 500 μM of uric acid (*purple*).

#

# 4. Temperature change of DT-functionalized AuNPs with different saliva dilution.


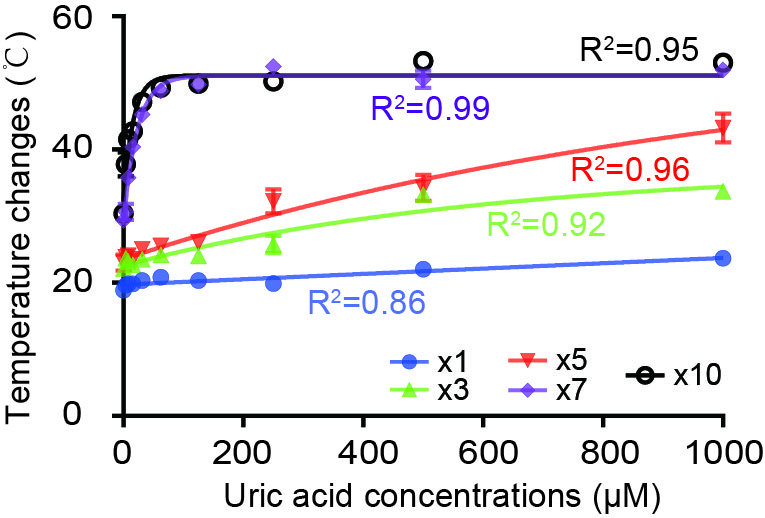


**Figure S4.** Photothermal detection of different levels of uric acid with different dilution ratios of saliva solutions (dilution ratios are ×1, ×3, ×5, ×7, and ×10).

# 5. Comparison of the performance between previously reported methods and this work for non-enzymatic uric acid determination.

**Table S1**. Comparison of the non-enzymatic uric acid detection methods in recent years.

| **Detection**  **principle** | **Material synthesis** | **Specificity** | **Detection range** | **Sample dilution** | **Detection method** | **Naked eye**  **detection** | **Ref.** |
| --- | --- | --- | --- | --- | --- | --- | --- |
| Pyrene-functionalized metal−organic framework | Complicate, at 120 °C for 72 hours | High | 1.4 – 1,000 µM | 100× | Fluorimetric | × | [[1](#_ENREF_1)] |
| Electrooxidation | Complicate, at 120 °C for  60 min | High | 8.8 – 53 µM | unknown | Electrochemical | × | [[2](#_ENREF_2)] |
| Molecular Imprinting Technologies | Complicate, at RT for  80 min | High | 5.0 – 160 μM | 5× | Electrochemical | × | [[3](#_ENREF_3)] |
| Excitation and quench of Tb and Eu complexes | Complicate, at 60ºC for  60 hours | High | 5.0 – 50 µM | 500× | Luminescence | × | [[4](#_ENREF_4)] |
| Coordination of antioxidants, Cu2+ and MOFs | Complicate, at 50ºC for  30 hours | Low | 20 – 100 µM | 1× | Fluorescence, colorimetric | √ | [[5](#_ENREF_5)] |
| 2-thiouracil tailored AuNPs | Simple, at RT for  40 min | High | 0.5 – 5  µM | 10× | Colorimetric | √ | [[6](#_ENREF_6)] |
| *DT-functionalized AuNPs system* | *Simple, at RT for*  *20 min* | *High* | *11.3 – 500 μM;*  *6.6 – 250 μM* | *7×* | *Colorimetric,*  *photothermal* | *√* | ***This work*** |

**References**

[1] R. Dalapati and S. Biswas, "A Pyrene-Functionalized Metal–Organic Framework for Nonenzymatic and Ratiometric Detection of Uric Acid in Biological Fluid via Conformational Change," *Inorganic chemistry,* vol. 58, pp. 5654-5663, 2019.

[2] C. Wang, J. Du, H. Wang, C. e. Zou, F. Jiang, P. Yang*, et al.*, "A facile electrochemical sensor based on reduced graphene oxide and Au nanoplates modified glassy carbon electrode for simultaneous detection of ascorbic acid, dopamine and uric acid," *Sensors and Actuators B: Chemical,* vol. 204, pp. 302-309, 2014.

[3] N. Li, C. Nan, X. Mei, Y. Sun, H. Feng, and Y. Li, "Electrochemical sensor based on dual-template molecularly imprinted polymer and nanoporous gold leaf modified electrode for simultaneous determination of dopamine and uric acid," *Microchimica Acta,* vol. 187, pp. 1-10, 2020.

[4] R. A. Poole, F. Kielar, S. L. Richardson, P. A. Stenson, and D. Parker, "A ratiometric and non-enzymatic luminescence assay for uric acid: differential quenching of lanthanide excited states by anti-oxidants," *Chemical communications,* pp. 4084-4086, 2006.

[5] X. Wei, J. Chen, X. Zhang, Z. Zhu, H. Liu, X. Wang*, et al.*, "Organic Framework@ Coordination Polymer Core-Shell Composites as Dual-Modal probe for Fluorescence and Colorimetric Analysis of Total Antioxidant Level in Saliva," *Sensors and Actuators B: Chemical,* vol. 347, p. 130588, 2021.

[6] R. K. Bera, A. Anoop, and C. R. Raj, "Enzyme-free colorimetric assay of serum uric acid," *Chemical Communications,* vol. 47, pp. 11498-11500, 2011.
